# Supplementary material for: The Evolution and Characterization of the RNA Interference Pathways in Lophotrochozoa
Source: Genome Biol Evol. 2024 May 7;16(5):evae098. doi: 10.1093/gbe/evae098 (PMC11114477; doi:10.1093/gbe/evae098)
Supplement: evae098_Supplementary_Data [file evae098_supplementary_data.zip › Supplementary_materials.zip!/selected_miRNAs/NoGe_selected_miRNAs/novel_miRNA_families_pdf_files/NMRB01000231.1_Notospermus_geniculatus_strain_Ushimado_scaffold231,_whole_genome_shotgun_sequence_7813.pdf]

Provisional ID	:	NMRB01000231.1_Notospermus_geniculatus_strain_Ushimado_scaffold231,_whole_genome_shotgun_sequence_7813
Score total	:	1721
Score for star read(s)	:	3.9
Score for read counts	:	1712.1
Score for mfe	:	1.9
Score for randfold	:	0
Score for cons. seed	:	3
Total read count	:	3370
Mature read count	:	3164
Loop read count	:	0
Star read count	:	206

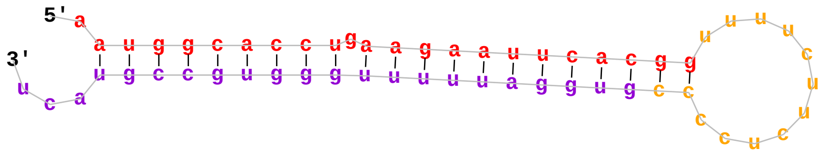

Genome coordinates: NMRB01000231.1 448590-448648

pre-miRNA sequence:
AATGGCACCTGAAGAATTCACGGAATGGCACCTGAAGAATTCACGGTTTTCTTCTCCCCGTGGATTTTTGGGTGCCGTACT

Mature sequence: AATGGCACCTGAAGAATTCACGG

BLAST results for the pre-miRNA sequence against the *Lineus longissimus* genome assembly:

Scaffold	Start	Stop	Identity	e-value
OU342999.1 1	1849517	1849539	100.000	0.001

```
Nge_novel_miRNA                    AATGGCACCTGAAGAATTCACGG
Llo_genome                         AATGGCACCTGAAGAATTCACGG
```
